# Psychometric properties of the Czech version of the Stigma Scale of Epilepsy

**DOI:** 10.1371/journal.pone.0195225

**Published:** 2018-03-29

**Authors:** Dana Brabcová, Jiří Kohout, Daniel Potužák, Barbora Beňová, Pavel Kršek

**Affiliations:** 1 Department of Psychology, Faculty of Education, University of West Bohemia, Plzen, Czech Republic; 2 Department of Physics, Faculty of Education, University of West Bohemia, Plzen, Czech Republic; 3 Department of Child Neurology, 2nd Faculty of Medicine, University Hospital Motol, Praha, Czech Republic; Johns Hopkins Hospital, UNITED STATES

## Abstract

Although significant attention has been devoted to analyzing stigma associated with epilepsy, there is still a significant lack of valid and reliable instruments. We aimed to validate the 23-item Czech version of the Stigma Scale of Epilepsy (SSE; originally developed in Brazil), which has been used to evaluate epilepsy-related stigma in the general population. Verification of the SSE questionnaire was carried out in a group of 207 students aged 15–18 years of whom none had epilepsy. These students completed the SSE twice in a period of 3–6 months as part of standard test-retest evaluation practice. The instrument exhibits good psychometric properties including internal consistency higher than in the original version (Cronbach’s alpha of 0.856 here compared with 0.81 reported in Brazil) and acceptable test-retest reliability. Using exploratory factor analysis (not provided for the original version), four factors were identified and corresponding subscales were described and interpreted. Two items did not fit into the structure and were eliminated. Confirmatory factor analysis was used to propose and verify the hierarchical 4-factor structure of the Czech version of SSE confirming the existence of a common factor corresponding to stigma. The results showed that the Czech version of SSE has good psychometric properties and can be used in further research and clinical practice.

## Introduction

Epilepsy has been associated with significant stigmatization since ancient times [1]. Although around 70% of people with epilepsy (PWE) respond to treatment and are seizure-free, stigmatizing attitudes regarding epilepsy are prevalent regardless of seizure control [2, 3]. Theories of stigma are usually based on the work of Goffman who defined the term stigma as an attribute which is socially discrediting in a particular way [4]. A detailed overview of theories of stigma may be found in one of the review articles [5,6,7].

Instruments able to accurately measure stigmatization are needed for effective stigma-reducing interventions [8]. A recent literature review identified only a few such interventions [9]. This may be partly due to the lack of consensus on how to measure stigma and to the corresponding lack of appropriate instruments, especially for the public [10]. Fernandes developed and validated for use in Brazil the 24-item Stigma Scale of Epilepsy (SSE) which utilizes the same questions to measure both stigma in PWE and epilepsy-related stigmatization in the public [11,12,13]. The scale exhibited high internal consistency, and was used in an intervention study focused on students in Brazil [14]. The factorial structure of the SSE was not investigated. Elafros et al. validated SSE on PWE in Zambia [10]. They used exploratory and confirmatory item response theories and compared the latent traits assessed by the SSE to those assessed by Jacoby’s Stigma Scale (JSS) described in Ref. 15. They found that SSE yielded in contrast to JSS two latent traits—the first reflected difficulties faced by PWE and the second reflected emotions associated with epilepsy [10]. As yet, no study has focused in detail on the psychometric properties of the SSE when used on people without epilepsy. Thus, the aim of this study is to validate the Czech version of the SSE for use on the public and to investigate its factorial structure.

## Materials and methods

### Procedure and respondents

Firstly, the SSE questionnaire measuring epilepsy-related stigma was translated from English into Czech in accordance with rules for the translation of research tools (i.e. two independent translators, a panel of experts checking both translations and determining a final version with respect to the nuances of the Czech language, and a translation of the final version back into English and comparison with the original, etc.). Preliminary research was conducted in a focus group of five students discussing the topic in order to confirm that our target group of students aged 15–18 years could understand the questionnaire fully. One item regarding sexual life of PWE was omitted due to inappropriate remarks of one of the younger students involved in the preliminary research. This resulted in a 23-item Czech version of the questionnaire. Nine randomly selected classes from three high schools in Pilsen, Czech Republic, were involved in the study (the participants of the aforementioned preliminary research were from other school and were not included anymore). The study was approved by authorities responsible for ethics in research at the University of West Bohemia, and was prepared in co-operation with principals of three high schools in Plzen, Czech Republic. The study was conducted in accordance with the 1964 Helsinki Declaration and its later amendments and comparable ethical standards. The parents of respondents younger than 16 years provided informed consent with the participation of their children in the research study. Informed consent has been also obtained from the participants older than 16 years. The only inclusion criterion was that none of the respondents had epilepsy according to the records of the particular school (according to Czech law, the caregivers are obliged to inform the school about the health status of their children and our experience show that an overwhelming majority of the parents of the children with epilepsy in the Czech Republic really do it). One of the authors of the study (D.P.) was responsible for arranging the research in the given classes and instructing the students how to complete the questionnaire. The participants had been informed in detail about the research and voluntary agreed with their participation in this fully anonymous study. However, due to the necessary pairing of test-retest data, respondents were asked to sign their questionnaires with an easy-to-remember nickname. After a period of 3–6 months, respondents were retested using the same questionnaire. The basic characteristics of the sample of respondents are shown in Table 1. As evident from the table, there were no significant differences between the initial testing and retesting. Approximately 25% of respondents knew a person with epilepsy (typically a relative, friend and/or former classmate from elementary school), while a number of respondents mentioned an experience with a dog having this condition.

**Table 1:** Basic characteristics of respondents.

Characteristic	First testing (n = 207)	Retesting (n = 169)	P-values for the tests of difference between the first testing and retesting
Gender			0.848 (computed using the two-sample z-test for the difference between the proportions)
Male	73 (35.3%)	58 (34.3%)	
Female	134 (64.7%)	111 (65.7%)	
Age	16.2±1.0^a^ years (range: 15–18 years)	16.1±0.8 years^b^ (range: 15–18 years)	0.055 (computed using the two-sample t-test)
Personal knowledge of a person with epilepsy			0.840 (computed using the two-sample z-test for the difference between the proportions)
Yes	53 (25.6%)	44 (26.0%)	
No	154 (74.4%)	125 (74.0%)	

^a^ average ± standard deviation

^b^ The age for retesting group is given at first testing (i.e. the average age of subgroup participating in the retest was 16.1 years at the first testing and thus almost the same as the corresponding age of the whole sample).

### Analysis

We used MS Excel with inbuilt accessory XLSTAT. In addition to classic descriptive statistics, we used correlation analysis, Cronbach’s alpha to determine the internal consistency of the questionnaire and exploratory factor analysis (EFA) using the method of principal axis factoring. The EFA assumptions (sample size, factorability, linearity and normality; for details see [15]) were tested and none of them were violated. We also carried out confirmatory factor analysis (CFA) using software LISREL 9.2. The test–retest reliability of the questionnaire was evaluated using intraclass correlation coefficients proposed by Fisher [16]. Results for the tests of normality and independence for the correlation coefficients were given with P-values. A test P-value of less than 0.05 was considered statistically significant. The same is true for parametric tests such as two-sample t-test and one-way Analysis Of Variance (ANOVA) which were used for comparison of mean values for two or more groups. Data underlying the findings presented in the manuscript are provided in the S1 Dataset.

## Results

### Descriptive statistics, normality and reliability

The sum score of SSE items on a scale of 0–100 points was computed as described in the original article on SSE [13]. The average value for the first testing was 46.17 and standard deviation was 12.98. No statistically significant differences were found either between the individual schools involved or between the classes within the schools (the P-value of the one-way ANOVA was significantly higher than 0.05 in all cases; see S1 Dataset for the details). It suggests that the distribution of the average stigmatization seems to be quite homogenous across the individual schools and classes. The sample skewness and kurtosis were 0.178 and -0.160 suggesting normally distributed data. It was subsequently confirmed by the Shapiro-Wilk test with the corresponding P-value of 0.554. The Cronbach’s alpha computed from polychoric correlation coefficients was 0.856 indicating very good internal consistency. To quantify test-retest reliability, an intraclass correlation coefficient of 0.51 was obtained from the sum score. According to the guidelines proposed by Cicchetti, it suggests fair test-retest reliability [17].

### Exploratory factor analysis (EFA)

**Fig 1:**
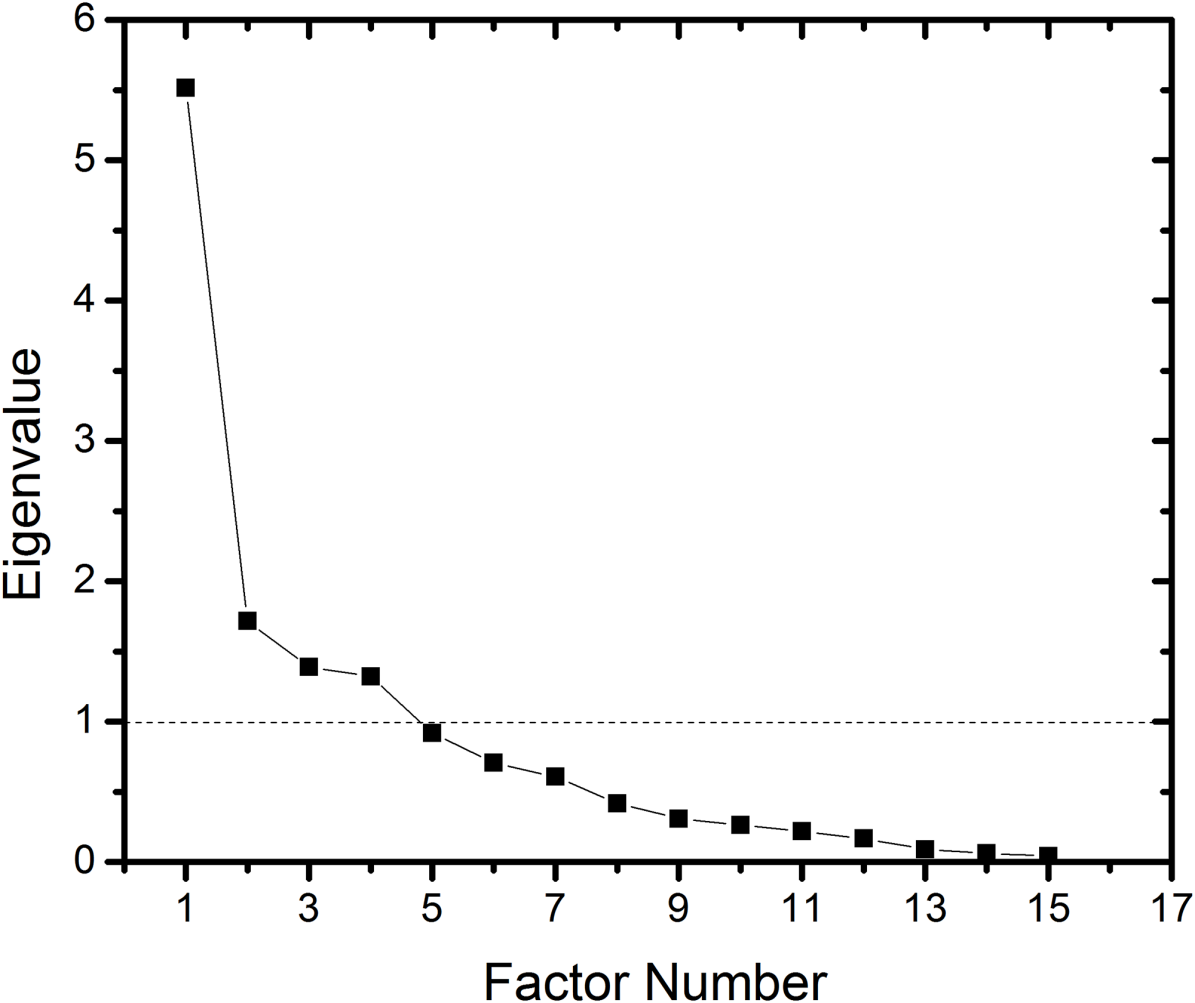
Scree plot for SSE exploratory factor analysis.

EFA based on polychoric correlations was performed and the number of factors was not restricted because no information about factorial structure of the original questionnaire was known. The particular parameters of the analysis were established based on the evaluation described in our previous study [18]. It was shown (Fig 1) that eigenvalues higher than 1 were found for the first 4 factors; however, the value for 5th factor was close to 1.

Hence, we rotated 1, 2, 3, 4 and 5 factors to obtain the most accurate questionnaire structure. Rotation was performed using the Varimax method and confirmed using the Oblimin method more suitable for correlated factors. No relevant differences were observed between both methods suggesting stability of the factor structure. Results were inconclusive upon the rotation of 1, 2, 3 and 5 factors (see S1 Dataset). In contrast, rotation of 4 factors led to unambiguous results. Table 2 shows factor loadings for the 4-factor solution. Items 1 and 4a were found to be problematic due to their factor loads of less than 0.4 indicating these items were not anchored well in the questionnaire structure [10]. Thus, these items were eliminated from the analysis.

**Table 2:** Factor loadings for the preferred 4-factor model.

Question/ item content	Factor 1 loadings	Factor 2 loadings	Factor 3 loadings	Factor 4 loadings	Subscale (properties)
*Q1*: *Do you think that PWE feel able to control their own epilepsy*?					
1	0.017	-0.037	-0.094	**0.366**	Not included in the model
*Q2*: *How would you feel when you see an epileptic seizure*?					
2a: Scare/Shock^c^	0.048	**0.698**	0.046	0.045	2 (54.7±20.8^a^, α = 0.709^b^)
2b: Fear	0.096	**0.788**	-0.021	0.041	
2c: Sadness	0.249	**0.451**	0.370	-0.060	
2d: Pity	-0.004	**0.470**	0.370	0.030	
*Q3*: *Which difficulties do you think PWE have in their daily lives*?					
3a: Relationships	**0.549**	-0.011	0.354	0.379	1
3b: Work	0.092	-0.016	**0.749**	0.121	3 (51.7±20.7^a^, α = 0.784^b^)
3c: School	0.299	0.013	**0.653**	0.421	
3d: Friendship	**0.607**	-0.100	0.126	0.259	1 (37.4±18.7^a^, α = 0.818^b^)
3f: Emotional	**0.524**	0.061	0.063	0.421	
3g: Prejudice	**0.460**	-0.019	0.164	0.291	
*Q4*: *How do you think that PWE feel*?					
4a: Worried	0.054	**0.267**	0.160	0.190	Not included in the model
4b: Dependent	0.262	0.123	-0.097	**0.406**	4 (44.5±17.5^a^, α = 0.733^b^)
4c: Incapable	0.101	-0.035	0.178	**0.680**	
4d: Fearful	0.145	0.337	0.105	**0.491**	
4e: Depressed	0.213	0.023	0.348	**0.529**	
4f: Ashamed	0.239	0.081	0.230	**0.506**	
4g: The same as those without epilepsy	-0.056	0.244	0.141	**0.425**	
*Q5*: *In your opinion*, *the prejudice in epilepsy will be related to*?					
5a: Relationships	**0.670**	0.089	0.261	0.155	1
5b: Marriage	**0.892**	0.115	0.142	-0.001	
5c: Work	0.183	0.066	**0.642**	-0.060	3
5d: School	0.182	0.188	**0.625**	0.237	
5e: Family	**0.540**	0.112	-0.073	-0.043	1

Note. Factor loadings in bold denote the dominant factor for the individual item.

^a^ average ± standard deviation

^b^ the value of Cronbach’s alpha computed from polychoric correlations.

^c^see Discussion for details regarding the translation from Czech to English

### Confirmatory factor analysis (CFA)

We conducted CFA to determine whether the data fit the preferred 4-factor model, and to compare it with the factor models tested and rejected during EFA. The results of CFA are usually interpreted using indices of fit. Thus, we followed the same procedure as in our previous study [19] and the recommendations to state at least one absolute fit index, one relative fit index and one index expressing model parsimony [20].

For maximum simplicity and clarity, the following limiting conditions were imposed on all models:

- Each item was saturated with one factor for which exploratory factor analysis found the largest load;
- No error covariances between items were considered;
- Correlations between all factors in all models were taken into account.

Root-mean square error approximation (RMSEA) was selected from a number of absolute indices. In RMSEA, a smaller value indicates better data-model fit, lower values than 0.08 suggest acceptable fit. Comparative fit index (CFI) was chosen from a number of relative indices. This index has a range of 0–1, with higher values corresponding to better data-model fit. Parsimony goodness-of-fit index (PGFI) was selected from indices reflecting model parsimony. Lower values correspond to higher level of parsimony of the model and the model that produces the lowest value is the most superior. In order to directly compare the individual models, we computed Akaike information criterion (AIC) which is an estimator of the relative quality of models for given set of data. The lower value of AIC, the higher is the relative likelihood of the given model [21].

Besides the five aforementioned models, we tested also a hierarchical model assuming that the four factors of first order suggested by exploratory analysis are all linked to second-order factor which would represent stigma as a common factor.

The results (Table 3) show that the best results were obtained from the hierarchical model exhibiting the lowest value of RMSEA, the highest value of CFI as well as the lowest value of AIC. It suggests that the second-order factor corresponding to overall stigma should be included. High value of the Pearson correlation coefficient of 0.968 between the corresponding factor score and the sum score of the questionnaire indicates that the sum score is a reasonable approximation to the underlying factor score and thus represents well this common factor.

**Table 3:** Properties of the factorial models tested using confirmatory factor analysis and the corresponding fit indices.

	One-factor model	Two-factor model	Three-factor model	Four-factor model	Five-factor model	Hierarchical four-factor model
Number of items	21	21	21	21	21	21
Number of first-order factors	1	2	3	4	5	4
Number of correlations between factors	0	1	3	6	10	4^c^
Number of parameters in the model	42	43	45	48	52	46
Number of parameters in the saturated model	231	231	231	231	231	231
Number of degrees of freedom	189	188	186	183	179	185
Maximum likelihood ratio chi-square value	600.16 (<0.0001^a^)	550.26 (<0.0001)	489.07 (<0.0001)	387.49 (<0.0001)	368.23 (<0.0001)	374.27 (<0.0001)
Root-mean square error approximation (RMSEA)	0.111 (0.101; 0.120)^b^	0.104 (0.095; 0.114)	0.084 (0.075; 0.093)	0.074 (0.065; 0.083)	0.074 (0.065; 0.083)	0.073 (0.065; 0.082)
Comparative fit index (CFI)	0.581	0.629	0.702	0.764	0.762	0.770
Parsimony goodness-of-fit index (PGFI)	0.623	0.630	0.674	0.665	0.685	0.662
Akaike information criterion (AIC)	3603.52	3555.62	3474.08	3417.38	3416.40	3413.61

^a^ P-value for the test of hypothesis that the original correlation matrix is the same as the correlation matrix derived from the model.

^b^ 95% confidence interval for the index RMSEA

^c^number of paths between the factor of second order and four factors of first order (correlations between the factors of first order were not included in the model)

### Factor interpretation and subscales

Based on theoretical and content analyses, the interpretation of factors given in Table 2 was recommended as follows:

1. The response to epilepsy in personal life
2. The emotional reaction of the public to epilepsy
3. The impact of epilepsy on work and school
4. The emotional perspective of individuals with epilepsy

Subsequently, we determined the descriptive characteristics of the subscales created from the factors. The results (Table 2) confirmed the high internal consistency of all subscales. Correlations between the subscales were positive and statistically significant, being in the range of 0.24–0.43.

### Effect of personal knowledge of someone with epilepsy

In order to evaluate possible effect of personal knowledge of a person with epilepsy on the level of stigmatization, we carried out a comparison using two-sample t-test for the sum score as well as for all subscales. The results are summarized in Table 4. It can be seen that the average sum score of stigmatization as well as two out of four subscale scores (subscales 1 and 4) are significantly lower for the participants who know someone with epilepsy. This finding is in accordance with the literature and support validity of the questionnaire.

**Table 4:** Effect of personal knowledge of someone with epilepsy on stigma (based on the first testing).

	Group 1 –participants knowing someone with epilepsy (n = 53)	Group 2 –participants not knowing someone with epilepsy (n = 154)	P-value of the two-sample t-test
Subscale 1	31.41±20.45^a^	39.35±17.66	**0.014**
Subscale 2	58.81±23.42	53.28±19.80	0.130
Subscale 3	48.08±19.63	52.85±20.99	0.140
Subscale 4	38.14±17.40	46.67±17.11	**0.003**
Sum score	42.45±14.00	47.41±12.41	**0.025**

^a^ average±standard deviation

Note. The P-values in bold denote cases when the corresponding null hypothesis was rejected at a significance level of 0.05.

## Discussion

EFA and CFA were used here to propose and verify the hierarchical 4-factor structure of the Czech version of SSE confirming the existence of a common factor. It was proved that it is meaningful to use the sum score of SSE established in the original version developed by Fernandes et al. Cronbach’s alpha as a measure of internal consistency was 0.856 here compared with the value of 0.81 from the original study in which the detailed structure of the questionnaire was not examined [13]. The test-retest reliability of the Czech version is not very high (the corresponding correlation coefficient of 0.51) in comparison with other instruments measuring attitudes. It could be caused by the relatively long period between the testing and retesting. Moreover, adolescents are more prone to significant change of attitudes than adults. It would be interesting to evaluate test-retest reliability among adults in further research.

SSE structure among PWE was for the first time studied by Elafros et al. as discussed in Introduction of this study [10]. Using item response theory they showed that SSE yielded two latent trails–the first reflected difficulties faced by PWE; the second reflected emotions associated with epilepsy. A comparison showed that the first of these factors corresponded well with our factors 1 and 3 concerned with response to epilepsy in personal life and the effect of epilepsy on school and work. The second factor identified in the aforementioned study corresponded to factors 2 and 4 focused on the emotional reaction of the public to epilepsy, and the emotional perspective of PWE. It seems that each of the two basic factors perceived by PWE was further divided into another two factors in the general population. One of these factors was intrapersonal and the other was interpersonal.

Items 1 and 4a proved to be problematic and were eliminated. In the case of item 1, it may have been due to the phrase “be able to manage their own epilepsy” which sounds quite vague and could be hard to grasp in Czech. The item was in fact excluded also from the recently validated Zambian version of the questionnaire [10]. The authors of the study from Zambia eliminated also item 2a due to high correlation (0.979) with item 2b [10]. This multicollinearity jeopardized model structure and thus item 2a was chosen for elimination due to its smaller factor load. In the present study, relatively high correlation of 0.666 was found between items 2a and 2b. However, this value was still far below 0.8 which is given as a limit in terms of multicollinearity. This could have been due to linguistic differences between Czech and English. It should be noted that authors of the study measuring using this questionnaire levels of stigma among relatives of PWE, health care professionals and students in Bolivia [22], translated item 2a using the word “shocked” instead of the word “scared” used in the original English written article [13]. We would prefer the same also for the translation from Czech to English.

Our study had some limitations. The results presented here should be generalized with caution because of the instrument was only validated with high school students in 9 classes in 3 schools. Even for the population of high school students the possibility to generalize is only limited even though the distribution of stigmatization across classes and schools appears to be relatively homogenous (see the section Descriptive statistics in Results).

The values of the fit indices are not so good even for the model hierarchical model preferred here. On the other hand, we used only simplified models and no error covariances between items were considered. Modification indices showed that the most important sources of misfit are just these missing error covariances particularly between the items saturated by factor 2 (the biggest decrease in chi-square, almost 60, would occur for the items 2a and 2b). Thus, the values of fit indices could be probably significantly improved with these modifications included in the preferred model. Adequate attention was not given to the convergent validity of the questionnaire. In our previous study focused on the effectiveness of interventions on reducing stigma, significant positive correlations between the SSE and a test measuring knowledge of epilepsy were observed suggesting convergent validity of the Czech version of SSE [23]. However, these variables should be examined in more detail. Another limitation is that we did not validate the questionnaire for PWE. Thus, it was not possible to accurately prove compliance or difference in factor structure in either group despite the comparison with [10] partially addressing this issue. No comparison with results from countries culturally closer to the Czech Republic is presented here because the SSE has not yet been validated in any European country. Despite these limitations, the study provided insight into the psychometric properties of the SSE questionnaire and showed that the Czech version of was suitable for use in the population not having epilepsy.

## Supporting information

S1 Dataset Data underlying the findings described in the manuscript. (XLS)

S1 Text Czech version of the questionnaire (in Czech). (DOC)
