# Supplementary material for: Psychometric properties of the Czech version of the Stigma Scale of Epilepsy
Source: PLoS One. 2018 Mar 29;13(3):e0195225. doi: 10.1371/journal.pone.0195225 (PMC5875900; doi:10.1371/journal.pone.0195225)
Supplement: S1 Text — (DOC) [file pone.0195225.s002.doc]

**STIGMA V SOUVISLOSTI S AUTISMEM**

Ráda bych Vás poprosila o vyplnění dotazníku, který je zaměřen na problematiku autismu

**Věk**:......................................... **Pohlaví:** muž žena

Jméno a příjmení……………………………………………………………………….Datum:....................................... Škola:.............................................................. Ročník:………………………………………….. Město:…………………………

Setkal/a jste se někdy dříve s pojmem **autismus**? (zakroužkuj správnou možnost) ANO NE

**Znáte osobně někoho s autismem? Pokud ano, koho**?

*Nyní si postupně přečtěte následující otázky a vždy* ***zakroužkujte*** *číslo, které nejlépe vyjadřuje Váš názor. Odpovídejte podle této hodnotící stupnice:*

*1 - vůbec ne 2 - trochu 3 - hodně 4 – zcela*

**Buďte prosím upřímní ve svých odpovědích. Pokud některé otázce nerozumíte nebo nevíte, jak odpovědět, nechte ji nevyplněnou. Děkuji za spolupráci.**

**1. Myslíte si, že osoby s autismem jsou schopny zvládat svou vlastní nemoc?**

1 2 3 4

**2. Jak byste se cítil/a, když byste viděl/a u autisty záchvat vzteku?**

a) vyděšeně 1 2 3 4 c) smutně 1 2 3 4
b) vystrašeně 1 2 3 4 d) soucitně 1 2 3 4

**3. Jaké potíže si myslíte, že mají autisté v každodenním životě?**

a) ve vztazích 1 2 3 4 b) v práci 1 2 3 4
c) ve škole 1 2 3 4 d) v přátelství 1 2 3 4
f) emocionální 1 2 3 4 g) s předsudky 1 2 3 4

**4. Jak si myslíte, že se cítí autisté?**

a) ustaraně 1 2 3 4 e) zahanbeně 1 2 3 4
b) závisle 1 2 3 4 f) depresivně 1 2 3 4
c) neschopně 1 2 3 4
d) ustrašeně 1 2 3 4
g) stejně jako bez autismu 1 2 3 4

**5. Podle Vašeho názoru budou předsudky o autismu souviset s:**

a) vztahy 1 2 3 4 d) školou 1 2 3 4
b) manželstvím 1 2 3 4 e) rodinou 1 2 3 4
c) prací 1 2 3 4

**Zkontrolujte, prosím, ještě jednou svoje odpovědi! Děkuji za vyplnění dotazníku!**
